# Supplementary material for: OpenBloodFlow: A User-Friendly OpenCV-Based Software Package for Blood Flow Velocity and Blood Cell Count Measurement for Fish Embryos
Source: Biology (Basel). 2022 Oct 8;11(10):1471. doi: 10.3390/biology11101471 (PMC9598615; doi:10.3390/biology11101471)
Supplement: Supplementary file 1 [file biology-11-01471-s001.zip › Supplementary File S2 Video File.pdf]

Video S1: Video to show blood flow before and after conducting image stabilization (https://www.youtube.com/watch?v=92UToolijvI) (accessed on 16 May 2022); Video S2: Tutorial video for introducing zebrafish blood flow measurement by OpenCV (https://www.youtube.com/watch?v=3MV9S7HtELw) (accessed on 16 May 2022); Video S3: Tutorial video for introducing medaka blood flow measurement by OpenCV (https://www.youtube.com/watch?v=PJqbWjmx7PM) (accessed on 16 May 2022). Video S4: Zebrafish blood flow alterations after exposure to PHZ at 0.15 ppm (https://www.youtube.com/watch?v=PwjjrvAdwMU) (accessed on 16 May 2022). Video S5: Japanese medaka blood flow at 10 days post-fertilization (dpf) (https://www.youtube.com/watch?v=ToQzKx7oaew) (accessed on 16 May 2022).
